# On-chip Fabrication of High Performance Nanostructured ZnO UV Detectors

**DOI:** 10.1038/srep08516

**Published:** 2015-02-17

**Authors:** Mohammad R. Alenezi, Simon J. Henley, S. R. P. Silva

**Affiliations:** 1 Nanoelectronics Center, Advanced Technology Institute, University of Surrey, UK; 2 College of Technological Studies, PAAET, Shuwaikh, Kuwait

## Abstract

Developing rationally controlled bottom-up device fabrication processes is essential for the achievement of high performance optimal devices. We report a controlled, seedless and site-selective hydrothermal technique to fabricate high-performance nanostructured ZnO UV-detectors directly on-chip. We demonstrate that by controlling the nanowire growth process, via tuning the experimental parameters such as the concentration of reactants and the growth time, and by introducing a refresh of the growth solution, the device structure efficiency can be enhanced to significantly improve its performance. The on-chip fabricated bridging nanosyringe ultraviolet detector demonstrates improved sensitivity (~10^5^), nanowatts detectability, and ultrafast response-time (90 ms) and recovery-time (210 ms). The improvement in response-time and recovery-time is attributed to the unique nanowire-nanowire junction barrier dominated resistance and the direct contact between ZnO and Au electrodes. Furthermore, the enhanced sensitivity and nanowatts detectability of the bridging nanosyringe device are due to the reduction in dimensionality and ultrahigh surface-to-volume ratio. This work paves the way toward low cost, large scale, low temperature, seedless and site-selective fabrication of high performance ZnO nanowire sensors on flexible and transparent substrates.

Zinc oxide (ZnO) nanostructures have been the center of extensive studies due to their potential applications for a wide range of devices such as laser diodes, light-emitting diodes, piezoelectric transducers and generators, gas sensors, and ultraviolet (UV) detectors^1^,^2^,^3^,^4^,^5^,^6^,^7^,^8^. Among these devices, UV detectors^6^,^7^,^8^ have been used in commercial and military applications. These uses include pollution monitoring, secure space communications, water purification, flame and missile plume detection, *etc*.^9^ Many reports, especially from the World Health Organization, have demonstrated evidence of harm associated with overexposure to UV. A series of different health effects have been identified, including skin cancer and malignant melanoma. Thus, for UV detecting materials to fit in these fields they need to have quick response and recovery, high sensitivity, good detectability, and high responsivity.

ZnO thin film based UV detectors usually require relatively long response time^10^. On the other hand, ZnO nanostructures demonstrate high photoresponse because of the increase in the surface-to-volume ratio and the active area reduced dimensionality^11^. Kind *et al*.^12^ reported the characteristics of a UV detector based on single nanowire (NW). In most of the reported studies the main aspect has been the investigation of the sensing mechanism to improve the sensitivity of the detector^13^. But, the speed of response and recovery of detectors have not been well investigated^14^.

From the device structure point of view, the fabrication of UV detectors based on NWs was carried out using one of two methods. The first method involves a pick-and-place process of a single NW and deposition of electrical contacts to individual NWs using electron-beam lithography, which is expensive and time consuming^13^. The other method involves multiple processes including growth, sonication, and distribution of NWs on substrates with prepatterned electrodes^15^. The complication of these fabrication processes is one of the key reasons behind the very limited practical applications of NW UV detectors.

ZnO NWs have been synthesized by different methods, such as physical vapor phase deposition^16^, metal organic chemical vapor deposition (MOCVD)^17^ and the low temperature hydrothermal method^2^,^3^,^5^. Compared to methods that require high operation temperatures and suffer from the limitation of using certain inorganic substrates only, the hydrothermal approach has been more attractive because of the wider range of substrates that can be used to grow the NWs at reasonable cost and the possible large scale production. In the majority of the reports on the hydrothermal synthesis of ZnO NWs, a pre-deposited seed layer is necessary for the nucleation^18^. Nevertheless, this may not be appropriate for many applications because it affects the contact between the NWs and the substrate.

Here, we report a controlled seedless and site-selective hydrothermal technique to fabricate high performance ZnO NW UV detectors on-chip. We demonstrate that by controlling the NWs growth, via tuning the experimental parameters, the device structure efficiency can be increased to significantly enhance its performance (sensitivity, detectability, response-time, and recovery-time). Using this on-chip fabrication technique, two different UV detectors are fabricated on flexible and transparent substrates, the bridging NW (BNW) and bridging nanosyringe (BNS) device, and are compared with two other detectors with different device structures, the NW array (NWA) and single NW (SNW) device.

## Experimental

Si/SiO_2_ and PET substrates with pre-patterned Au electrodes were used in this work. The growth solution consisted of zinc nitrate hexahydrate (Zn(NO_3_)_2_.6H_2_O, 98%, reagent grade, Sigma Aldrich), hexamethylenetetramine (HMTA), ammonium hydroxide (NH_3_.H_2_O, 30% wt%, reagent grade, Sigma Aldrich), and polyethylenimine (PEI) (end-capped, molecular weight 800 g/mol LS, Sigma Aldrich).

To fabricate the BNW devices, substrates with prepatterned Au electrodes were face-down in a vial containing zinc nitrate hexahydrate (25 mM), HMTA (12.5 mM), and ammonium hydroxide (0.35 M) as an initial growth stage where the vial was heated in an oven at 85°C for 45 min. The secondary growth stage was started by adding PEI (5 mM) to the growth solution in the vial and ended after 3 h at the same temperature. After the growth is terminated, the substrate was washed with hot water to remove PEI and annealed at 150°C for 10 min after that.

In the fabrication process of BNS devices, the initial stage was the same as in the BNWs device case. However, in the second stage the refreshing solution used was a mixture of zinc nitrate hexahydrate (10 mM), HMTA (5 mM), ammonium hydroxide (0.35 M), and PEI (10 mM).

ZnO NWA and SNW UV detectors were fabricated for comparison. For ZnO NWA devices, the prepatterned substrate was spin coated with 5 mM zinc acetate dehydrate Zn(CH_3_COO)_2_.2H_2_O solution in ethanol at 1000 rpm for 30 s. The spin-cast layer on the substrate was cured on a hot plate 150°C for 5 min to stabilize the film structure. The spin coating and curing processes were repeated five times in order to obtain a uniform film, which served as the seeding layer. Afterwards, the film was thermally annealed at 350°C for 30 min, and then allowed to cool down. The thermal decomposition (of the zinc acetate) created ZnO nanocrystals on the substrate that act as a seed layer for subsequent ZnO array growth. The precursor solution for the hydrothermal reaction consists of Zn(NO_3_)_2_.6H_2_O (25 mM), HMTA (12.5 mM), PEI, (5 mM) and NH_3_.H_2_O (0.35 M). The seeded substrate was then placed in a vial that contains (15 mL) of the growth solution. The vial was covered and then placed in an oven and heated at 85°C for 7 h. after which the growth is terminated, the substrate then rinsed with DI water and dried in air at 150°C for 30 min.

PEI is soluble in hot water (70–75°C). Thus, all samples in our work were washed repeatedly with hot water after the termination of the growth process and left in refreshed hot water for 2 days to totally remove the adsorbed PEI from their surfaces.

SNWs devices were fabricated by drop casting a solution containing ZnO NWs on substrates with Au prepatterned electrodes. The substrate was annealed at 200°C for 2 h for better contact between the NWs and the electrodes.

The crystal structure of the as-prepared NWs were analyzed through powder X-ray diffraction (XRD) using a Panalytical X-pert diffractometer with CuKα radiation. The morphology and crystal structure of as-prepared products were observed using Philips XL-20 scanning electron microscope at 10 kV. Scanning transmission electron microscopy (STEM) and electron diffraction measurements were performed on a Hitachi HD2300A microscope operating at 200 kV. STEM samples were prepared by depositing a drop of diluted suspension of the NWs in ethanol on a carbon film coated copper grid.

The electrical characteristics of the fabricated devices were recorded using a probe station attached to a Keithley 4200 semiconductor analyzer. The excitation source for the UV detection properties was a UV lamp with maximum intensity at a wavelength of 365 nm. All the measurements were at a bias of 2 V.

## Results and Discussion

In the reactions of the classical HMTA assisted hydrothermal synthesis of ZnO NWs, formaldehyde and ammonia are produced as a result of the hydrolysis of HMTA during the NWs growth process^19^. This serves as a pH buffer by slow decomposing gradually to control the amount of ammonia, which provides OH^−^ by forming ammonium hydroxide. OH^−^ and Zn^2+^ are known to form a number of complex monomeric hydroxyl species^20^. By the dehydration of these hydroxyl species, solid ZnO nuclei are formed. Surface hydroxyl groups condensate with the zinc–hydroxyl complexes to proceed the growth of ZnO crystals^2^,^21^. Furthermore, the ability of HMTA and ammonia to coordinate to Zn^2+^ and control its concentration allows the species in solution to be kinetically controlled. The equilibrium of the growth reactions can be controlled by tuning the reaction parameters, such as growth solution concentration, growth temperature and growth period.

In this work, the ratio of zinc nitrate to HMTA in the solution was 2:1, causing the supersaturation degree to be very high with respect to ZnO or Zn(OH)_2_. This usually leads to the homogeneous nucleation and formation of ZnO solid in the solution. Additionally, these unwanted ZnO solids grown in the solution can easily contaminate the substrates, which degrades the performance of devices in many applications. However, introducing ammonium hydroxide into the reaction system can suppress the homogeneous nucleation significantly, since ammonia can coordinate to free Zn^2+^ and lower their concentration^19^,^22^. Then, the complexes buffer Zn^2+^ and release them gradually resulting in a lower degree of supersaturation. Alternatively, substrates can be seeded by coating them with ZnO thin film prior to the growth process. Thus, ZnO NWs can grow on the seeded substrates without the need of initial nucleation stage.

Based on the literature^19^,^22^, high concentrations of ammonium hydroxide could significantly slow the growth process because of the low degree of supersaturation. That has led many groups to use polyethyleneimine (PEI) in order to extend the length of the NWs by inhibiting the radial growth but allowing their axial growth and enhance the aspect ratio of the grown NWs^2^,^19^,^22^. It is known that the polymer chain of PEI can be absorbed onto the crystal facets formed in homogeneous nucleation process inhibiting any further growth along them. Thus, it is very difficult for clusters to grow and achieve the critical size that thermodynamically favors the growth of crystals. PEI has proven to be very effective in enhancing the aspect ratio of the ZnO NWs grown on substrates with large enough preexisting seeds.

**Figure 1:**
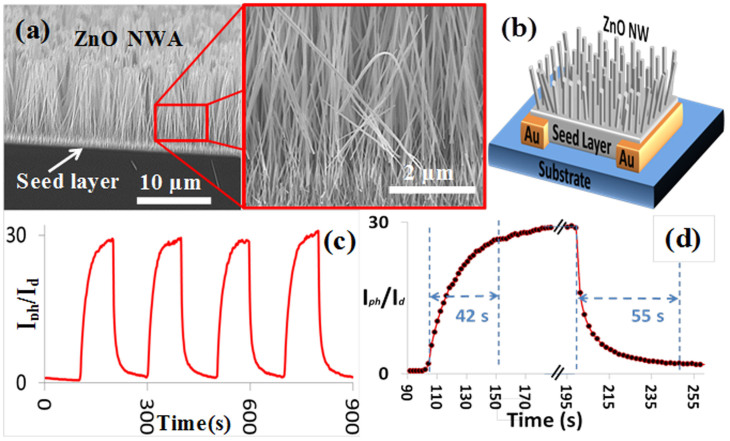
(a) SEM images, (b) schematic diagram, (c), and (d) the photoresponse characteristics of the ZnO NWA detector.

Applying this synthesis technique, ZnO NWA UV detectors were fabricated. Figure 1 shows SEM images, schematic, and photoresponse characteristics of a ZnO NWA grown on ZnO seeded Si/SiO_2_ substrate with prepatterned Au electrodes. The photosensitivity of this device, which is defined as the ratio of the photocurrent I*_ph_* to the dark current I*_d_*, is about 30. The response time and the recovery time are 42 and 55 s, respectively. In our experiments we tested many UV detectors with this type of device structure and in some cases the response-time and recovery-time were as long as several minutes. The conduction channel in this device structure is the seed layer beneath the NWs (polycrystalline thin film ~300–400 nm) that is connecting the two electrodes. Even though the photoresponse of the device is stable with high repeatability, the photosensitivity is low with long response-time and recovery-time. Moreover, its fabrication process involves a seed layer preparation which requires annealing at a temperature of around 400°C for at least 30 min, thus making it is unsuitable for flexible substrates.

**Figure 2:**
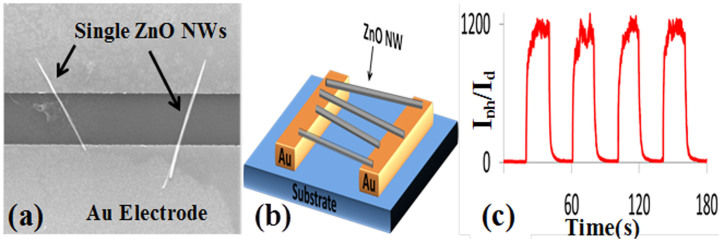
(a) An SEM image (2.5 μm space between the Au electrodes), (b) schematic diagram, and (c) the photoresponse characteristics of the ZnO SNW detector.

UV detectors with a different device structure were also fabricated based on single ZnO NWs grown using the same synthesis technique. An SEM image, schematic, and photoresponse characteristics of the ZnO SNW detectors are shown in figure 2. The photosensitivity of this device is about 1200 with a response-time and recovery-time of 11 and 15 s, respectively. The photosensitivity, of this detector is 40 times that of the NWA detector and it is 4 times faster in response and recovery. This improvement was expected due to the reduction in the dimensionality of the SNWs compared with the NWA and also the higher fields that can be applied across the two electrodes to give larger depleted active volumes. It is worth mentioning that this device can be fabricated using flexible substrates, but the fabrication process involves multiple steps since it requires the growth of the NWs on seeded rigid substrates first before transferring them to flexible substrates with prepatterned electrodes. Additionally, the size and number of the SNWs making the detector are difficult to control as it is apparent in the SEM image of the SNW detector in figure 2(b) where the two SNWs next to each other are different in dimensions and in their contact with the electrodes.

In order to enhance the UV detector sensitivity, detectability and speed of response and recovery, a more efficient device structure that can be fabricated on flexible and transparent substrates is required. In this case the device structure can be improved by (1) growing ZnO NWs directly on the electrodes without the seed layer (better contact), (2) introducing a depletion layer blocking transfer mechanism (NW-NW junction), (3) reducing the dimensionality of the NWs, and (4) controlling the number of the BNWs. To satisfy the above stated requirements, we modified this synthesis technique to become controlled, seedless and site-selective. A series of different experiments were performed in order to optimize the parameters of the growth process to achieve these goals.

**Figure 3:**
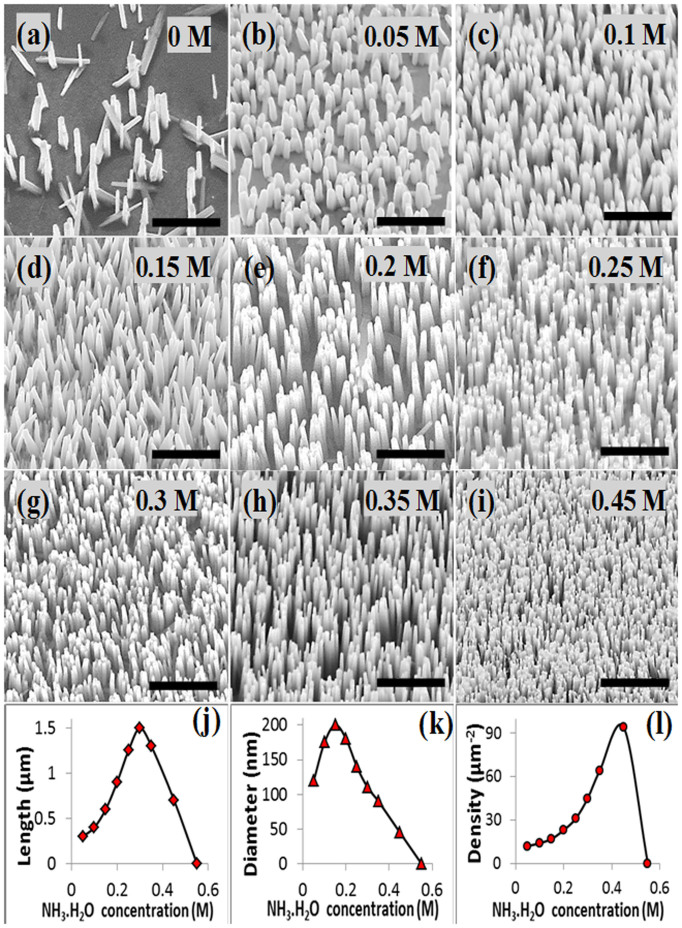
Ammonium hydroxide concentration influence on the morphology of ZnO NWs grown on seedless gold layer. (a)–(i) SEM images of NWs grown under Ammonium hydroxide concentrations of 0, 0.05, 0.1, 0.15, 0.2, 0.25, 0.3, 0.35, and 0.45, respectively. The scale bar is 2 μm. (j)–(l) Dependence relationship of length, diameter, and density of the NWs on concentration of Ammonium hydroxide.

First, we needed to modify the growth solution and the substrate material to be able to grow ZnO NWs without seeding the substrate. Figure 3 shows SEM images of ZnO NWs grown on Au coated Si/SiO_2_ substrates in a mixed solution of zinc nitrate (25 mM), HMTA (12.5 mM), and different concentrations of ammonium hydroxide at 90°C for 3 h. Without adding ammonium hydroxide, the result dispersed relatively large NWs with an average diameter of 300 ± 43 nm and length of about 1 ± 0.15 μm (figure 3(a)). In fact, most of the nutrient material has been consumed by nucleating homogeneously and found at the base of the vial after the growth. As a result, the grown NWs are dispersed.

The case became different once ammonium hydroxide was included in the growth solution. Figures 3(b)–(i) show SEM images of ZnO NWs grown seedlessly at ammonium hydroxide concentrations of 0.05, 0.1, 0.15, 0.2, 0.25, 0.3, 0.35, and 0.45 M, respectively. At a concentration level of 0.05 M of ammonium hydroxide, the number of nucleation sites on the substrate has improved significantly in comparison with the nucleation rate without ammonium hydroxide as evident in figure 3(b). The density of the NWs at this level was 12 ± 1.1 NW.μm^−2^ and the average diameter size was 120 ± 45 nm. Yet, the amount of NH^4+^ in this case was not enough to consume all of the zinc hydroxide leaving the solution in a state of turbidity. Consequently, at this low concentration level of ammonium hydroxide, the length of the NWs was relatively small around 300 nm.

Raising the concentration of ammonium hydroxide further to 0.1 M decreased the rate of Zn^2+^ release further and limited the available nutrient materials at the beginning of the growth process. As a result, homogeneous nucleation in the bulk solution was suppressed further encouraging the heterogeneous nucleation on the substrate and increasing the density, diameter size, and length of the NWs to 14 ± 1.25 NW.μm^−2^, 175 ± 32 nm, and 400 nm, respectively (figure 3(c)). At ammonium hydroxide concentration of 0.15 M, the density of the NWs increased slightly to 17 ± 0.95 NW.μm^−2^. The diameter size and length were also increased to 200 ± 39 nm and 600 nm, respectively (figure 3(d)).

Further increases in the concentration of ammonium hydroxide beyond this level caused the average diameter size of the NWs to decrease because the number of free Zn^2+^ at the beginning of the growth process was getting smaller while the density of nucleation sites was increasing. At the concentration level of 0.2 M, the average diameter size of the NWs was 180 ± 37 nm, and their length was 900 nm (figure 3(e)). The density was increased further with this increase in the concentration of ammonium hydroxide to 23 ± 1.3 NW.μm^−2^ as a result of the strongly encouraged heterogeneous nucleation on the substrate.

As the concentration of ammonium hydroxide was further increased to 0.25, 0.3, 0.35, and 0.45 M, the density of the NWs kept increasing and recorded the following values 31 ± 1, 45 ± 0.9, 64 ± 0.7, and 94 ± 1.2 NW.μm^−2^, respectively. The diameter size of the NWs on the other hand kept decreasing and the measured values are 140 ± 33, 110 ± 49, 90 ± 52, and 48 ± 31, respectively. The following length values of 1.25, 1.5, 1.3, and 0.7 μm were measured respectively for the same levels of ammonium hydroxide concentration.

To observe clearly and analyze the impact of ammonium hydroxide concentration on the morphology of the grown NWs, different areas of the substrates were analyzed and the average length, average diameter, and average density of the as-synthesized NWs were calculated and represented in the plots in figures, respectively.

The plots in figures 3(j)–(l) represent the average length, average diameter, and average density of the as-grown NWs as functions of the concentration of ammonium hydroxide, respectively. The length of the NWs increases with the increase in concentration of ammonium hydroxide from 0.05 to 0.25 M where the maximum value occurs, and then starts to decrease as shown in figure 3(j). For the average diameter of the NWs, the same trend is observed with the maximum value being at 0.15 M. For the ammonium hydroxide concentration range from 0.05 to 0.45 M, the density of the NWs kept increasing and the maximum was attained at 0.45 M. The effect of the increasing density of NWs on their diameter size and length is understood since the amount of nutrient is fixed in the solution and as a result increasing the nucleation sites leads to smaller NWs. By further increasing the concentration of ammonium hydroxide up to 0.55 M, the huge amount of NH_4_^+^ prevented any nucleation on the substrate and no NWs were grown.

It is clear that the results of the seedless growth processes of ZnO NWs with and without the use of ammonium hydroxide are different. The NWs produced without using ammonium hydroxide have larger dimensions, low density, and poor alignment (figure 3(a)). On the other hand, those produced with ammonium hydroxide have smaller dimensions, high density, and good alignment, which could be attributed to the different solution environments. Without ammonium hydroxide, there are many available Zn^2+^ ions, while using ammonium hydroxide limits them, which significantly lowers the rate of homogeneous nucleation and encourages the heterogeneous one. This eventually leads to a better growth without the need of seed layer^19^,^22^. Our results reported here provide guidelines on controlling the concentration of ammonium hydroxide to engineer the growth process of the ZnO NWs for the optimized density and dimensions.

**Figure 4:**
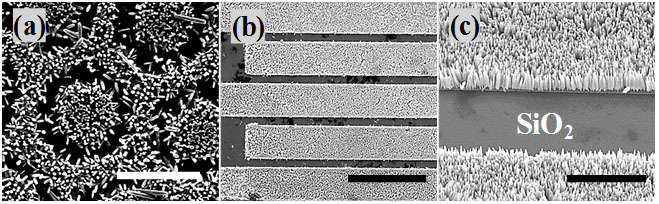
(a), (b), and (c) SEM images of the as-grown NWs on Si/SiO_2_ substrates with different prepatterned Au electrodes at different magnifications. (Scale bars in (a), (b), and (c) are 10, 50, and 5 μm, respectively).

Depending on the strong nucleation of this synthesis technique on Au surfaces, ZnO NWs were grown on substrates with different prepatterns of Au which are shown in figure 4. It is evident in these SEM images that ZnO NWs grew significantly on areas deposited with Au while no nucleation on silicon dioxide can be seen. These images show clearly that ZnO NWs can be site-selectively grown on Au surfaces of different shapes, opening the door for many potential applications of this growth technique including gas sensors, UV detectors, piezoelectronics, solar cells etc.

Once again, the density of ZnO NWs can be improved greatly by increasing the concentration of ammonium hydroxide, but the growth rate will be very low and the growth process would be long^19^. With the intention of enhancing the aspect ratio of the grown NWs, PEI was used to extend the length of the NWs by inhibiting the radial growth but allowing the axial growth. Despite all the great results achieved by using PEI in the seed layer assisted growth method, it was observed that introducing PEI in the seedless synthesis system at the beginning of the growth process and before initial nucleation stage disturbs the entire growth process. In the nucleation stage, PEI will encapsulate any formed nuclei. This observation has also been reported by another group^22^. Hence, in order to overcome this problem, PEI was introduced after the nucleation stage is over by refreshing the growth solution with a new one containing PEI.

**Figure 5:**
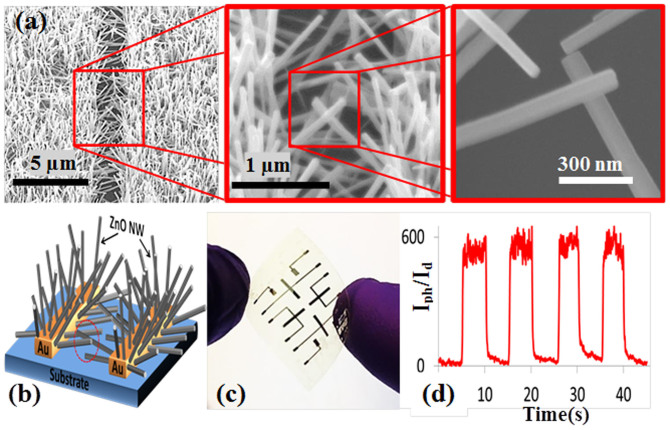
(a) SEM images, (b) schematic diagram, (c) image of the BNS device fabricated on flexible transparent substrate, and (d) the photoresponse characteristics of the ZnO BNW device.

Using the seedless hydrothermal synthesis technique to grow ZnO NWs directly and selectively on Au electrodes that are prepatterned on flexible and transparent substrates, we fabricated UV detectors with a different device structure. The detectors are based on bridging NWs (BNWs) from the two Au electrodes. SEM images at different magnifications, schematic, and a digital image of the BNW detector are shown in figure 5(a)–(c), respectively. From these images we can see that ZnO NWs are bridging both electrodes, forming a NW-NW junction as shown in the high magnification SEM image. The photoresponse characteristics of the BNWs detector are shown in figure 5(d). The photosensitivity is around 600, which is 20 and 0.5 times that of the NWA and the SNW detector, respectively. The response-time and the recovery-time of the BNW device are 250 and 340 ms, respectively. This device responds 168 and 44 times faster than the NWA and SNW device, respectively. The enhancement in the response-time and recovery-time is attributed to the more efficient device structure of the BNW detector with a junction between every two bridging NWs compared with NWA and SNW devices. The effect of the presence of these NW-NW junctions on the performance of the UV detectors will be discussed in more detail later.

The photosensitivity enhancement of the BNWs over the NWA device is again due to the low dimensionality of the conducting channels in the BNW device. On the other hand, even though they have the same dimensionality, the photosensitivity of the SNW device is a double that of the BNW device. The reason behind this is that the conducting channels in the BNW device are on top of each other and the ones that are below are not well exposed to the UV irradiation.

**Figure 6:**
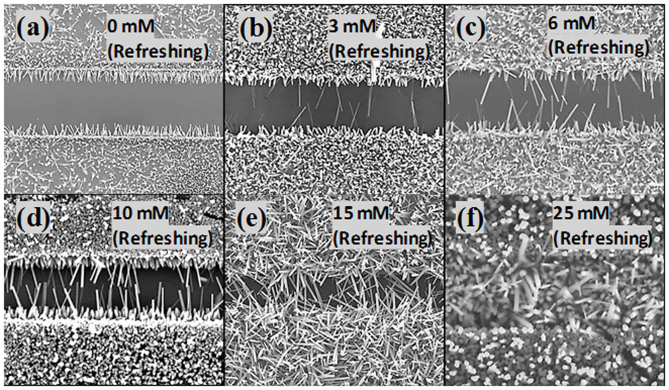
SEM images of ZnO NWs on Si/SiO_2_ substrates with prepatterned Au electrodes grown using a refreshing growth solution with a Zn(NO_3_)_2 _concentration of (a) 0 mM, (b) 3 mM, (c) 6 mM, (d) 10 mM, (e) 15 mM, and (f) 25 mM. (The space between the electrodes is 2.5 μm).

To avoid the disadvantage of the BNWs overlapping their density, diameter, and length must be controlled. In our experiments we found that the density, diameter, and length of the BNWs can be controlled by tuning the nutrient concentration and the amount of PEI added in the refreshing solution, and by varying the growth time. Figure 6 shows top view SEM images of ZnO NWs grown on prepatterned Au electrodes using growth solutions with different nutrient concentrations. The initial growth solution was a mixture of zinc nitrate (25 mM), HMTA (12.5 mM), and ammonium hydroxide (0.35 M). In the refreshing solution, a fixed amount of PEI (5 mM) was added and the concentration of NH_3_.H_2_O (0.35 M) was fixed in all cases, and only the concentration of Zn(NO_3_)_2_ and HMTA (maintaining the same ratio of 2:1) was changed. Therefore, in this set of experiments we only specify the concentration of Zn(NO_3_)_2_. When only PEI and NH_3_.H_2_O were added as a refreshing solution with no Zn(NO_3_)_2_ and HMTA, the growth process stopped completely and no growth occurred after adding refreshing solution due to the absence of the source materials (figure 6(a)). However, a low density growth was observed when a 3 mM of Zn(NO_3_)_2_ and 1.5 mM of HMTA were added as shown in figure 6(b). When the concentration of the nutrient in the refreshing growth solution was further increased to 6, 10, and then 15 mM the density was enhanced accordingly as evident in figures 6(c)–(e), respectively. By increasing the nutrient concentration up to 25 mM, the space between the two Au electrodes was completely blocked by the bridging ZnO NWs (figure 6(f)). These results show clearly that we can control the density of the BNWs, which is advantageous from device structure standpoint and makes the fabrication technique suitable for a wider range of applications.

**Figure 7:**
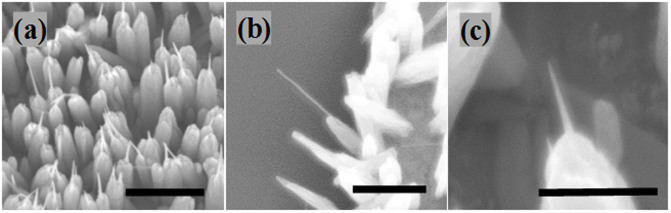
SEM image of (a) ZnO NS array (b) single ZnO NS, and (c) single ZnO NS at high magnification. (Scale bars in (a)–(c) are 500, 500 and 200 nm, respectively).

Furthermore, in a different set of experiments, we investigated the influence of PEI concentration in the refreshing solution on the grown NWs by fixing all other parameters and controlling the concentration of PEI only. We started by adding less than 3 mM of PEI with the refreshing solution and after 2 h the NWs length was extended from 200 to 300 nm with no significant change in the diameters (200–300 nm) and density of the NWs. When 5 mM of PEI were added, the length of the NWs increased from 200 to 600 nm with a limited effect on their diameters and density. Increasing the concentration of added PEI to 10 mM increased the length of the NWs from 200 to 800 nm and caused a dramatic reduction in the diameters of the NWs from 200 to ~30 nm (figure 7(a)). This significant change in the diameter size of the NWs after refreshing the growth solution transformed the NWs into syringe-like structures, nanosyringes (NSs) as shown in figure 7(b)–(c). Increasing the PEI concentration up to 15 mM led to a total encapsulation of the NWs and no further growth.

**Figure 8:**
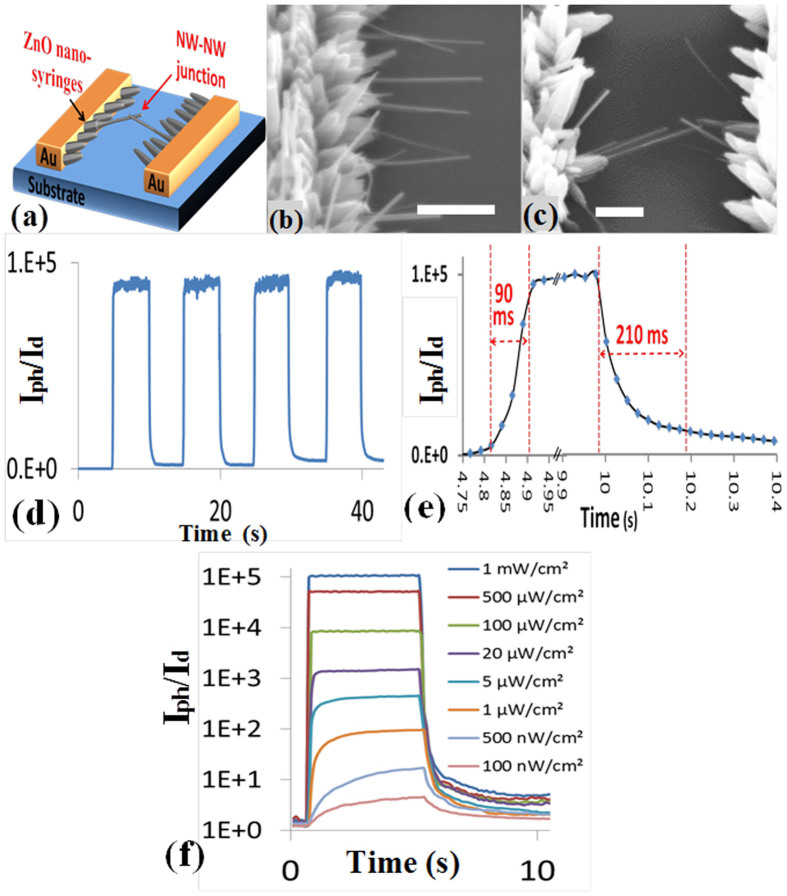
(a) A schematic diagram,(b), (c) SEM image (Scale bars are 500 nm), (d), (e) the photoresponse characteristics, and (f) the photoresponse in logarithmic scale at different UV intensities of the ZnO BNS device.

After gaining good control over the density, length, and diameter of the seedless and site-selective grown ZnO NWs bridging the two Au electrodes, a modified device structure based on BNSs was designed to overcome the limitations of all the previous devices. Figure 8 shows schematic, SEM images, photoresponse characteristics under multiple switching of the UV light, and the photoresponse under different UV light intensities of the BNS device. The photosensitivity of this detector is more than 10^5^ as shown in figure 8(d), which is 4 orders of magnitude higher than that of the NWA detector and 85 and 166 times that of the SNW and BNW detectors, respectively. The response-time and recovery-time of the BNS detector are 90 and 210 ms, respectively (figure 8(e)).

The detectability of the BNS device was also investigated. Figure 8(f) shows the photoresponse of the BNS detectors to UV intensities ranging from nano to milliwatts. The BNS detector was able to detect the UV light with an intensity as low as a 100 nW/cm^2^. It is also observed from the same plot that the photoresponse of the detector increases as the UV intensity increases. However, the response-time and recovery-time were found to increase with the increase in the UV light intensity. The response-times of the detector under UV irradiation with intensities of 0.1, 1, 100, 1000 μW/cm^2^ are 4.8, 3.5, 1.2, 0.2, 0.09 s, respectively.

The superiority in photosensitivity of BNS device over the other devices originates from the reduced dimensionality (20–40 nm in diameter) and high aspect ratio (~100) as well as the efficient device structure with a junction between every two BNSs and better contact with the electrodes compared with NWA and SNW devices.

**Figure 9:**
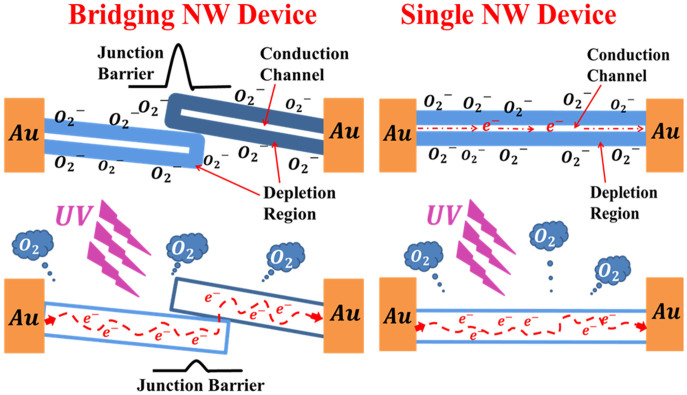
A schematic diagram depicting the carrier generation and transportation processes in the single- and bridging-NWs UV detectors.

The processes of generating and transporting carriers in the SNWs and BNWs devices are illustrated in the schematic diagram shown in the Scheme in figure 9. ZnO is an n-type semiconductor because the oxygen vacancies (V_O_) in it donate electrons to its conduction band^23^. When a single ZnO NW is in air, oxygen molecules will be adsorbed on its surface capturing electrons from its conduction band as oxygen ions (O^2−^). This will create a low-conductivity depletion layer near the surface of the NW. The negative charges at the surface creates band bending, and further extenuation of quantum confinement perpendicular to the NWs surface due to quantum size effects. As a result a surface potential barrier is generated. When ZnO NWs are irradiated with UV, electron-hole pairs are created and the negatively charged surface attracts holes and traps them.
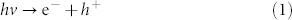
Oxygen ions neutralize these holes trapped at the surface and the result is many free electrons in the confined core.
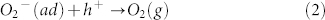
Therefore, UV excitation increases the number of free electrons in the NW and narrows the depletion layer. As a result, the current will gradually increase until saturation. When the UV irradiation to the ZnO NWs is stopped, holes recombine with electrons and oxygen molecules are readsorbed on the ZnO NW surface by capturing electrons which increases its resistance^23^,^24^,^25^.

The conductivity of the BNW and BNS detectors is affected by a mechanism that does not exist in the SNW detectors. The conduction channels in the BNW and BNS detectors include NW-NW junctions. Electrons must get over the junction barrier to pass from one NW to another. These barriers are formed by the surface depletion layers. The conductance of the ZnO NW can be expressed as:
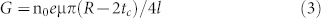
where n_0_ is the free charge carrier density, μ is the mobility of the charge carriers, *R* represents the diameter of the NW, *t*_c_ is the thickness of the surface charge region, *l* is the length between the two electrodes. The thickness of the depletion region *t*_c_ is given by:
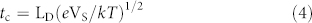
where V_S_ is the adsorbate-induced band bending while L_D_ is the Debye length. The Debye length in turn is given by:
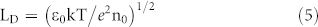
Where ε is the relative dielectric permittivity of the nanostructure^26^. The depletion regions in each NW act as barriers that hinder the electron transfer from one NW to another, since the current has to pass through these depletion zones of connected NWs. Therefore, when the diameters of the NW exceed the Debye length, the predominant modulator of charge transport in BNWs devices is the NW-NW junction.

The improvement in time of response and recovery in the BNW and BNS detectors can be ascribed to the charge transport controlled by the NW-NW junction barrier. Diffusion and readsorption of oxygen to deplete the NW channel is a relatively slow process^27^,^28^. The charge recovery time will be relatively long if the device conductance is controlled by the NW conductance only such as the SNW and NWA devices. On the other hand, the conductance in the BNW and BNS detectors is dominated by the NW-NW junction barriers that can be considered as two back-to-back Schottky barriers. There is an exponential relationship between the tunnel current across the junction barrier and the barrier height. Hence, the current is very sensitive to small changes in the barrier. When the UV irradiation is stopped, the recombination of electrons and holes would increase the effective barrier as a result of the significant reduction in carrier density. The dominant effect of the junction barrier would lead to a fast current decay. The photo-induced barrier height modulation is typically much faster than the oxygen diffusion process^29^.

### Conclusions

In conclusion, we provide a facile and cost effective hydrothermal synthesis technique to fabricate high performance ZnO nanostructured sensors. Rational control over the morphology, structure, and position was demonstrated by tuning the experimental parameters of the growth process. The BNS UV detector demonstrated improved photosensitivity (~10^5^), nanowatt detectability, and ultrafast response-time (90 ms) and recovery-time (210 ms) compared with typical devices. The origin of the enhanced response-time and recovery-time was ascribed to the unique NW-NW junction barrier dominated resistance and the direct contact between ZnO and the Au. The improvement in photosensitivity and detectability were due to the reduction in dimensionality and ultrahigh surface-to-volume ratio compared to the other tested structures. The technique presented in this report can be extended to fabricate more practical devices using other metal oxides at low cost and in large scale production.

## Author Contributions

M.R.A. conducted all the experimental work and prepared figures. M.R.A. and S.R.P.S. wrote the main manuscript text. S.R.P.S., S.J.H. and M.R.A. conceived the idea to conduct the work proposed and reviewed the manuscript.
